# Mechanical and Electrical Characteristics of WB_2_ Synthesized at High Pressure and High Temperature

**DOI:** 10.3390/ma13051212

**Published:** 2020-03-08

**Authors:** Changchun Wang, Lele Song, Yupeng Xie

**Affiliations:** 1 College of Science, Jilin Institute of Chemical Technology, Jilin 132022, China; xieyupeng1981@163.com; 2 Institute of Atomic and Molecular Physics, Jilin University, Changchun 130012, China; songlele_322@126.com

**Keywords:** hard material, electrical resistivity, tungsten diboride, Vickers hardness

## Abstract

Single-phase tungsten diboride (WB_2_) was synthesized at high pressure and high temperature. The different grain sizes ranging from 300 nm to 3 µm were successfully obtained in WB_2_ by controlling the experimental conditions. The effects of grain size on hardness and resistivity properties were investigated. The Vickers hardness of WB_2_ was modulated with grain size. The maximum asymptotic Vickers hardness is 25.5 GPa for WB_2_ with a grain size of 300 nm which is a 10% increase compared to WB_2_ with a grain size of 3 µm. The optimal electrical resistivity of WB_2_ was 10^−7^ Ωm with the biggest grain size of 3 µm, which is ascribed to low grain boundary density. The superior properties of hardness and electrical resistivity demonstrate that WB_2_ should be a new functional hard material replacing WC which is widely used in industrial production.

## 1. Introduction

Recently, transition metal borides (TMBs) have been attracting considerable attention for their unique physicochemical properties, such as high melting point, hardness, electrical conductivity, wear resistance, thermal conductivity and chemical inertness, etc. [1,2,3,4]. Due to their outstanding properties, TMB ceramics have been widely used in particular environments, such as abrasive, corrosion-resistant, conductive and electrode materials [5,6,7,8]. In TMBs, tungsten diboride (WB_2_) was predicted to have a very high hardness ranging from 36 to 40 GPa and good conductive properties by first-principle calculation [3,9]. However, there is a paucity of reports about the hardness and conductive property of WB_2_. These reports about WB_2_ are ascribed to the fact that bulk WB_2_ is hard to synthesize at ambient temperature by traditional methods, such as self-propagating combustion synthesis [10], chemical vapor deposition (CVD) [11], and spark plasma sintering [7,12]. Solid state WB_2_ shows lower self-diffusion coefficients and so it is difficult to facilitate its densification in the process of sintering using powder as precursor. Even in high temperature sintering, WB_2_ will not exhibit any shrinkage [13]. So, high quality bulk WB_2_ samples cannot be synthesized by traditional methods so far. Therefore, an exploration of the mechanical properties of WB_2_ has been hindered. 

In this work, single-phase WB_2_ was prepared under high pressure and high temperature. We measured its electrical resistivity and hardness. It is worth noting that we can modulate the hardness and electrical resistivity of WB_2_ by controlling the experimental conditions. In addition, we also find that WB_2_ shows excellent conductive properties. The high hardness and low electrical resistivity demonstrate that WB_2_ can be used as conductor working under extreme conditions.

## 2. Materials and Methods

In this study, powdery tungsten (200 mesh, 99.95% in purity) and amorphous boron powder (500 mesh, 99.99% in purity) were used as the starting materials. Powder mixtures which contained W:B atomic ratios of 1:2 and 1:2.2 were mixed in an agate mortar for 3.5 h. The mixed powders were then cold-pressed into cylindrical samples of 4 mm in diameter and 3 mm in height. Finally, the samples were prepared in a cubic anvil HPHT apparatus (SPD-6 × 600, Xianyang, China) at a target temperature (in this study temperatures were 1600 °C, 1700 °C, 1800 °C and 1900 °C, respectively), and a pressure of 5.2 GPa for a holding time of 15 min. The detailed process is as follows: firstly, the samples were put into the HPHT apparatus and the pressure was then raised to 5.2 GPa; secondly, the samples were heated to the target temperature in 15 seconds and the samples were kept at the target pressure and target temperature for 15 min; finally, the samples were cooled naturally to room temperature and removed from the apparatus. The phase analyses of the as-synthesized samples were examined by an X-ray diffractometer (XRD) using Cu-Ka (l = 1.5404 Å) radiation in a Rigaku D/max-2500 X-ray diffractometer (Japan). The morphological properties of the samples were analyzed with a scanning electron microscope (JEOL JSM-6700F, Japan). The electrical resistivity of WB_2_ was measured using a four-point probe system. The Vickers microhardness measurements were performed by a Micro-Hardness Tester (HV-1000ZDT), and the applied load P and Vickers hardness (H_V_) were determined using equation:

H_V_ = 1854.4P/d^2^, (1)

Here, d is the mean of the two diagonals of the indent and the holding time under the peak load was 15 s. The drainage method was used to measure density. The density ρ was determined using equation:

ρ = m/v(2)

Here, m is the mass of the sample and V is the volume of boiling water discharged from the sample.

## 3. Results and Discussion

**Figure 1:**
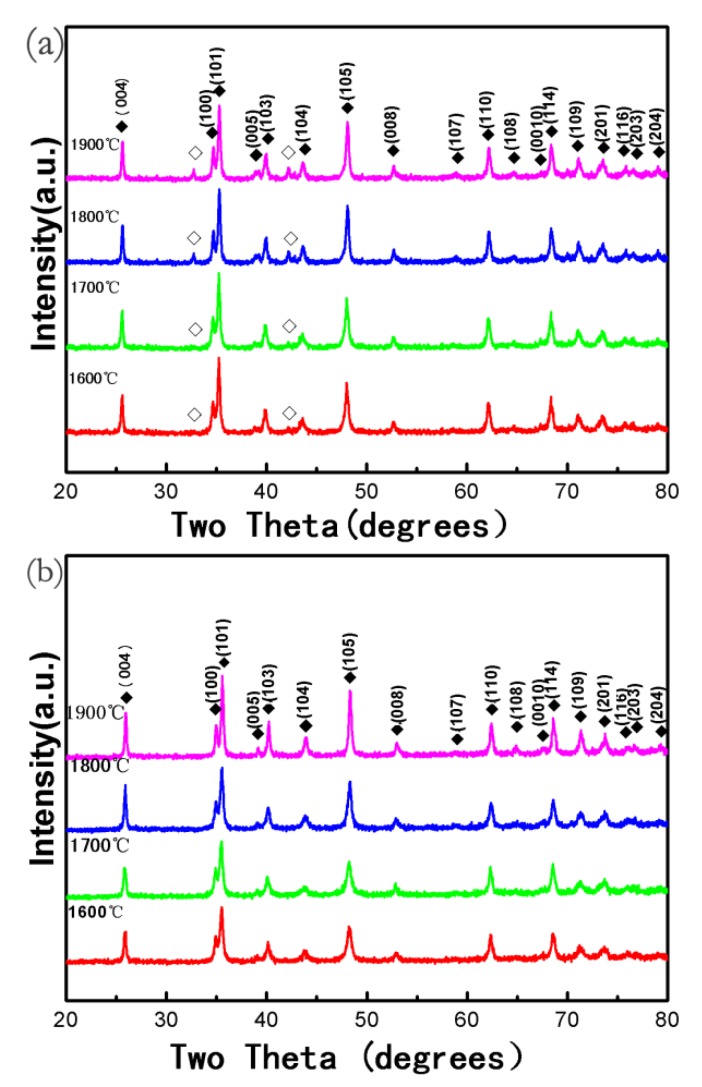
XRD patterns of the samples fabricated (**a**) W/B atomic ratios of 1/2 at 5.2 GPa and different temperatures (1600–1900 °C) for 15 min, (**b**) W/B atomic ratios of 1/2.2 at 5.2 GPa and different temperatures (1600–1900 °C) for 15 min. Solid rhombus represents tungsten diboride (WB_2_). Hollow rhombus represents WB.

In order to synthesize the stoichiometric phase in pure WB_2_, powder mixtures that contained W/B atomic ratios of 1/2 were used as a precursor. We found that single phase WB_2_ without impurity is hard to synthesize. Figure 1a shows the XRD patterns of WB_2_ samples synthesized at 5.2 GPa and temperatures of 1600 °C to 1900 °C for 15 min. We can detect that WB_2_ space group: P6_3_/mmc; ICSD Number: 023716 is the major phase and coexists with WB space group: I41/amd; ICSD Number: 024281). The phenomenon that stoichiometric phase pure WB_2_ cannot be synthesized by using a stoichiometric W/B atomic ratios precursor, often appears in TMBs synthesis, and may be caused by an inadequate proportion of B. In the process of mixing the precursor, boron powder was easily absorbed by mortar and spread in the air. The loss of boron samples led to the precursor exhibiting a nonstoichiometric ratio. In order to synthesize phase pure WB_2_, the proportion of B in the precursor should be increased. When we changed the atomic ratios of W/B to 1/2.2, phase pure WB_2_ was synthesized easily. Figure 1b shows the XRD patterns of WB_2_ samples synthesized using the mixed precursor with W/B atomic ratios of 1/2.2 at 5.2 GPa and temperatures of 1600 °C to 1900 °C for 15 min. All the materials were confirmed to be phase pure.

**Figure 2:**
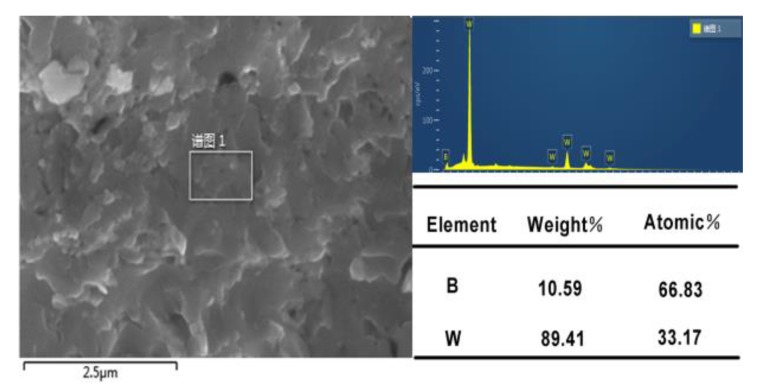
EDS images of as-synthesized compound fabricated at 5.2 GPa and different temperatures (1600–1900 °C) for 15 min using W powder and B powder (with W/B atomic ratios of 1/2.2) as precursor.

The stoichiometry ratio of the compound was checked by EDS and the results are shown in Figure 2. The picture is one of the images we have taken. The atomic ratio of W/B shown in Figure 2 is the average value of ten measurements. In measurement, we chose different parts to measure. The molar ratio of W and B for the compound was approximately confirmed to be 1:2.0. So, single phase WB_2_ without impurity cannot be synthesized with W/B atomic ratios of 1/2 because the proportion of B is inadequate.

**Figure 3:**
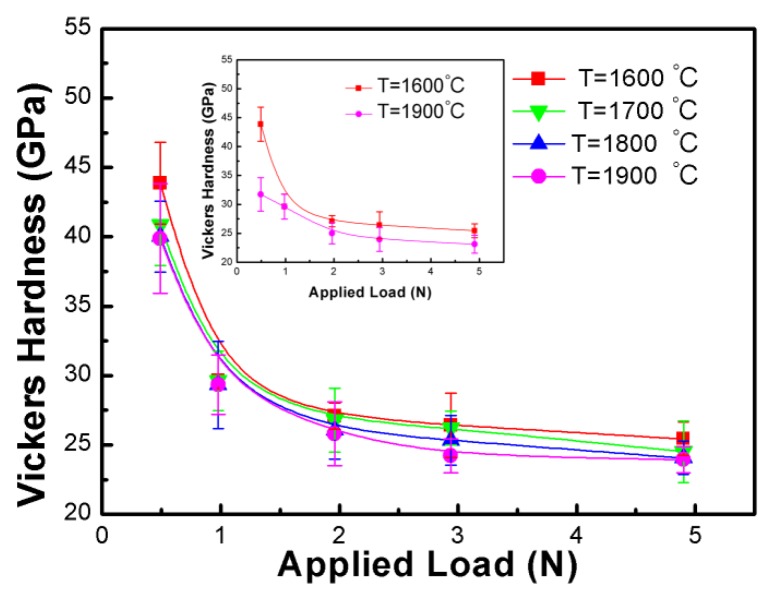
The asymptotic-hardness of WB_2_ synthesized at temperatures of 1600 °C to 1900 °C with the pressure at 5.2 GPa for 15 min.

The well-sintered bulk samples possessed high densification. We performed Vickers hardness (Hv) measurement after polishing. The measurement details are described in the experiment section. The obtained hardness results are shown in Figure 3. The hardness decreased as the loading pressure increased. The load-dependent Vickers hardness data for WB_2_ are shown in Figure 3. A maximum measured hardness of 43.875 GPa at 0.49 N was obtained and this value was higher than the threshold value of superhard material [14]. It is reported that hardness in the asymptotic-hardness region may occur because it is closer to the intrinsic hardness [15,16]. As shown in Figure 3, the asymptotic-hardness of WB_2_ synthesized at temperatures of 1600 °C is about 25.5 GPa. This is higher than that of WC (22.0 GPa) [17], which is most widely used as a hard material in industrial applications, under the same applied load. The high hardness of WB_2_ suggests that WB_2_ is a promising candidate for use as a hard material.

**Figure 4:**
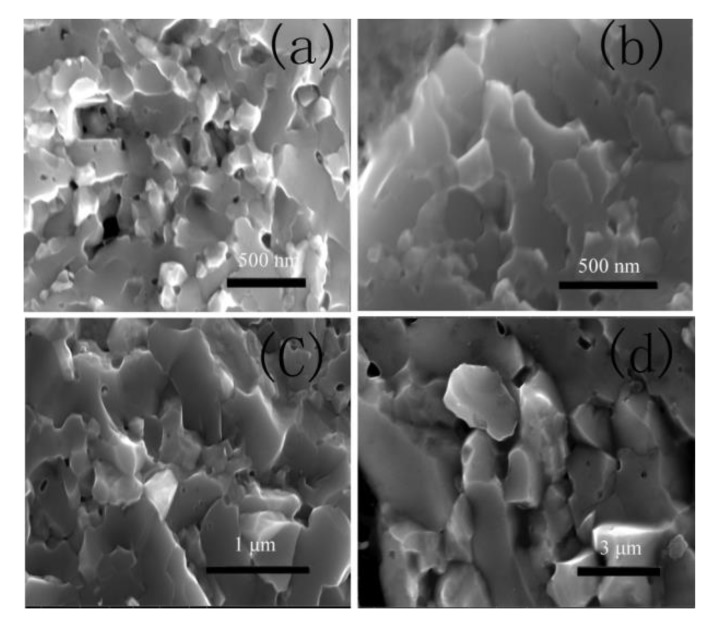
SEM results of as as-synthesized sample. (**a**), (**b**), (**c**) and (**d**) show the SEM results of WB_2_ synthesized at 5.2 GPa for 15 min, with different temperatures of 1600 °C, 1700 °C, 1800 °C, 1900 °C, respectively.

It is worth noting that the maximum asymptotic Vickers hardness of WB_2_ synthesized at 1600 °C increased by 10% compared to WB_2_ synthesized at 1900 °C. It is known that increasing temperature could improve the densification of a sample, and the densification could affect the hardness. So, the hardness should increase with the synthesis temperature increasing at a certain pressure. However, in our result, the hardness of WB_2_ synthesized at a low temperature was higher than that of WB_2_ synthesized at a high temperature. In order to find why our conclusion was contrary to other findings, SEM tests were performed. SEM images of phase-pure WB_2_ synthesized at different temperatures are shown in Figure 4. As is shown in the SEM images, WB_2_ has different grain sizes in the range of 200 nm−3 μm. As temperature accelerates grain growth, it was found that the grain size increased as the temperature increased at a certain pressure. In the conventional synthesis method, temperature could improve the densification of sample ceramics. The denseness of a sample has an obvious effect on its hardness, and the hardness increases as the temperature increases. That is to say, the denseness of sample is the main factor in affecting hardness. In the HPHT method, the denseness of sample does not change obviously. So, the denseness of WB_2_ is not the main factor in affecting hardness. It is reported that grain size can affect hardness. Smaller grains have more grain boundaries which can impede the propagation of stress during the hardness test. This increase in hardness with a decrease in crystal size is known as the Hall-Petch effect [18,19]. In our study, the largest grain size was more than tenfold that of the smallest grain size. So, the grain size of WB_2_ has an obvious effect on hardness. This requires more force to make the crystal slip, and so the smaller the grain size, the greater the yield limit of the material. However, in this work, the grain size was not at the nanoscale. So, WB_2_ cannot have a rapid increase in hardness. It can be concluded from the above that the grain size had an obvious influence on the hardness of WB_2_ synthesized in our experiment.

**Figure 5:**
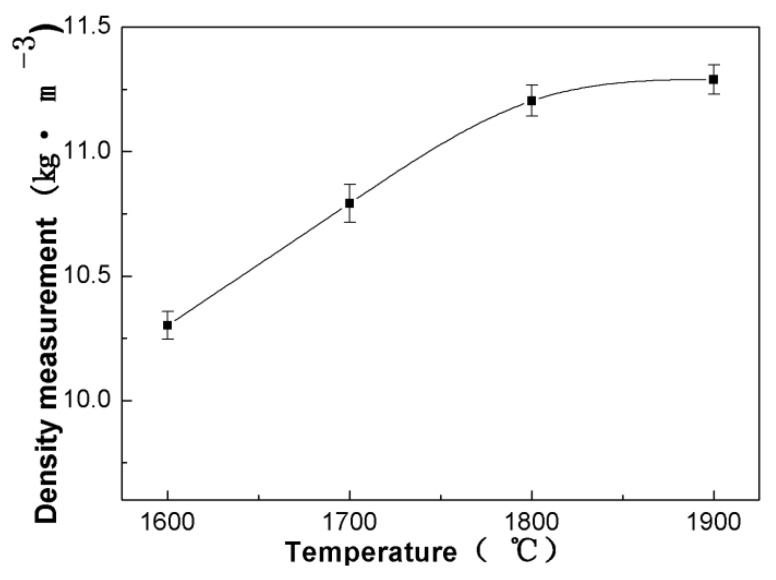
Density of WB_2_ samples versus temperature at pressures of 5.2 GPa for 15 min.

In order to further confirm that the changes in the hardness of WB_2_ were caused by grain size but not density, density measurements were performed on the as-synthesized samples. Density results are shown in Figure 5. The density increased with the increase in the temperature in the entire temperature range. Considering that materials with a high density always show high hardness, the high hardness of WB_2_ synthesized at low temperatures could be caused by small grain size.

**Figure 6:**
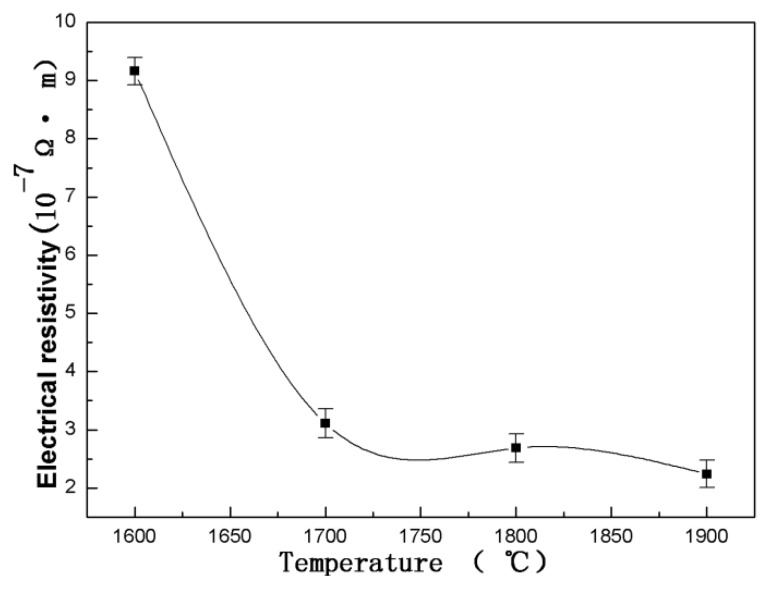
The variation of electrical resistivity with temperature for WB_2_ synthesized at 5.2 GPa and temperatures of 1600 °C to 1900 °C for 15 min.

The Electrical resistivity of WB_2_ synthesized at different temperatures was measured, and the results are shown in Figure 6. As the synthesized temperature increased, the electrical resistivity of WB_2_ decreased from 9.164 × 10^−7^ Ωm to 2.349 × 10^−7^ Ωm. According to the electron scattering theory, the electrical conductivity is supposed to be approximately proportional to the grain size [20]. As the volume fraction of the interface in crystalline materials is roughly inversely proportional to the grain size, the dependence of residual resistivity on grain size correlated with that of the interfacial volume fraction. In Figure 4, we show that the grain size increases with the temperature in the entire temperature range. This result can explain why the electrical resistivity decreased while the temperature increased.

Furthermore, from the analysis of the density measurement results, we found that the density increased concurrent with the increased temperature in the entire temperature range. As has been demonstrated, density can influence resistivity. So the electrical resistivity is not only determined by the single condition of grain size. It can be concluded from the above results that the high conductive properties of a material are not only dependent on its high density, but also on a large grain size.

## 4. Conclusions

In this work, different grain size samples of WB_2_ were synthesized through a high pressure and high temperature reaction sintering method. Through an analysis of density, the use of a scanning electron microscope and electrical resistivity measurements, we found that both density and grain size had a direct connection with electrical resistivity. The Vickers indentation test showed that the Vickers hardness of WB_2_ decreased as the grain size grew larger. The maximum asymptotic Vickers hardness is 25.5 GPa. Comparatively, WC has a hardness of 22.0 GPa, which is lower than that of WB_2_. Results indicate that WB_2_ would be a new functional hard material.
